# Supplementary material for: Current-induced switching of proximity-induced ferromagnetic surface states in a topological insulator
Source: Nat Commun. 2021 Mar 3;12:1404. doi: 10.1038/s41467-021-21672-9 (PMC7930265; doi:10.1038/s41467-021-21672-9)
Supplement: Supplementary file 1 — Supplementary Information [file 41467_2021_21672_MOESM1_ESM.pdf]

Supplementary Information for
Current-induced switching of proximity-induced ferromagnetic surface states in a topological insulator

Masataka Mogi[1,2,5]*, Kenji Yasuda[1,5], Reika Fujimura[1], Ryutaro Yoshimi[2], Naoki Ogawa[1,2], Atsushi Tsukazaki[3], Minoru Kawamura[2], Kei S. Takahashi[2], Masashi Kawasaki[1,2], and Yoshinori Tokura[1,2,4]*

[1] *Department of Applied Physics and Quantum Phase Electronics Center (QPEC), University of Tokyo, Bunkyo-ku, Tokyo 113-8656, Japan.*

[2] *RIKEN Center for Emergent Matter Science (CEMS), Wako, Saitama 351-0198, Japan.*

[3] *Institute for Materials Research, Tohoku University, Sendai, Miyagi 980-8577, Japan.*

[4] *Tokyo College, University of Tokyo, Bunkyo-ku, Tokyo 113-8656, Japan.*

[5] *Present address: Department of Physics, Massachusetts Institute of Technology, Cambridge, Massachusetts 02139, USA.*

**Corresponding author. e-mail: mogi@mit.edu (M.M.); tokura@riken.jp (Y.T.)*

Supplementary Note 1 | Elemental distribution in a CGT/BST heterostructure.

We investigated cross-sectional scanning transmission electron microscopy (TEM) and energy dispersive x-ray spectroscopy on a CGT/BST heterostructure in our prior publication[1]. In the scanning TEM, we observe a highly sharp interface without interfacial mixing between the CGT and BST layers (please see Fig. 1 of Ref. 1). Also, in Supplementary Fig. 1b-e, we show the EDX elemental mapping in a CGT/BST/CGT heterostructure. As seen in the elemental distribution along the growth direction as averaged over the lateral direction (Supplementary Fig. 1f), Cr and Ge atoms are well localized in the CGT layer and their amounts are almost equivalent, being consistent with the $Cr_2Ge_2Te_6$ chemical composition, while Bi atoms locate only in the BST layer region. Furthermore, we used X-ray and polarized neutron reflectometry on the same heterostructure (please see Fig. 2 of Ref. 1). These macroscopic measurements strongly support the microscopic TEM/EDX results.

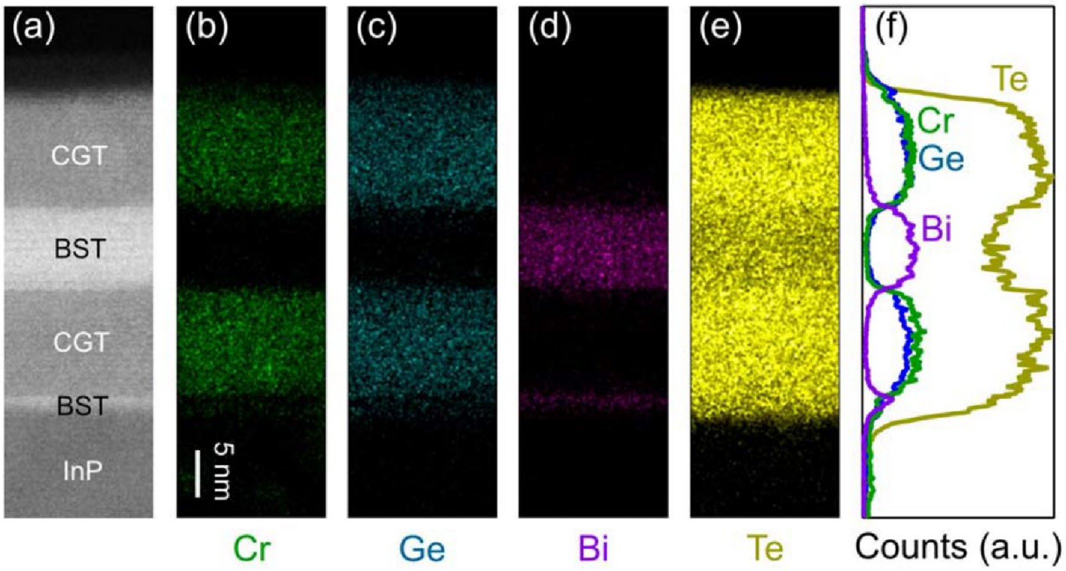

Supplementary Fig. 1 | TEM/EDX measurements in a CGT/BST/CGT heterostructure. a, Scanning TEM image corresponding to the EDX scan area. **b-e**, Elemental distribution maps for each element, Cr (**b**), Ge (**c**), Bi (**d**), and Te (**e**). **f**, Line profiles of Cr, Ge, Bi, and Te. Reprinted figure with permission from Ref. 1 Copyright 2019 by the American Physical Society.

Supplementary Note 2 | Additional transport data of CGT/BST bilayers.

We investigated the CGT thickness (t_{CGT}) dependence of transport in CGT/BST (x = 0.5) bilayers. As shown in Supplementary Fig. 2a, the change of t_{CGT} does not affect their sheet resistance R_{xx}, which may rule out possible effects of electronic structure changes in the bilayers on the thickness dependence of the magnetization switching (Fig. 2 in the main text). In addition, we compare R_{xx} of the CGT/BST bilayers with that of the BST single layer (Supplementary Fig. 2b). They show insulating behaviours while the BST single layer exhibits a metallic behaviour. We raise two possible reasons for the different temperature trends: (1) Holes are slightly transferred from the adjacent CGT layer to the BST layer and then the chemical potential approaches the Dirac point since the chemical potential of the BST single layer (Sb fraction x = 0.5) lies above the Dirac point, and (2) the exchange interaction between the CGT and BST layers opens the magnetic gap in the Dirac surface state, which removes the antilocalization property of the gapless Dirac surface state.

Next, by varying the Bi/Sb ratio (x), the Fermi level (E_F) position is effectively controlled. Supplementary Fig. 2b displays the temperature (T) dependence of R_{xx} in the CGT (3.5 nm)/$(Bi_{1-x}Sb_x)_2Te_3$ (6 nm) bilayer films with various Sb fraction x. When x = 0.3, 0.5, and 0.7, the T dependence shows the semiconducting behaviour, implying the E_F lies in the TI bulk gap. On the other hand, when x = 0 and 1, the metallic behaviour with the lower R_{xx} appears, indicating the E_F intersects the TI bulk states. We note that the R_{xx} for all the heterostructures are much smaller than the CGT single layer (> 1 MΩ below 50 K) as shown in Fig. 1e of the main text.

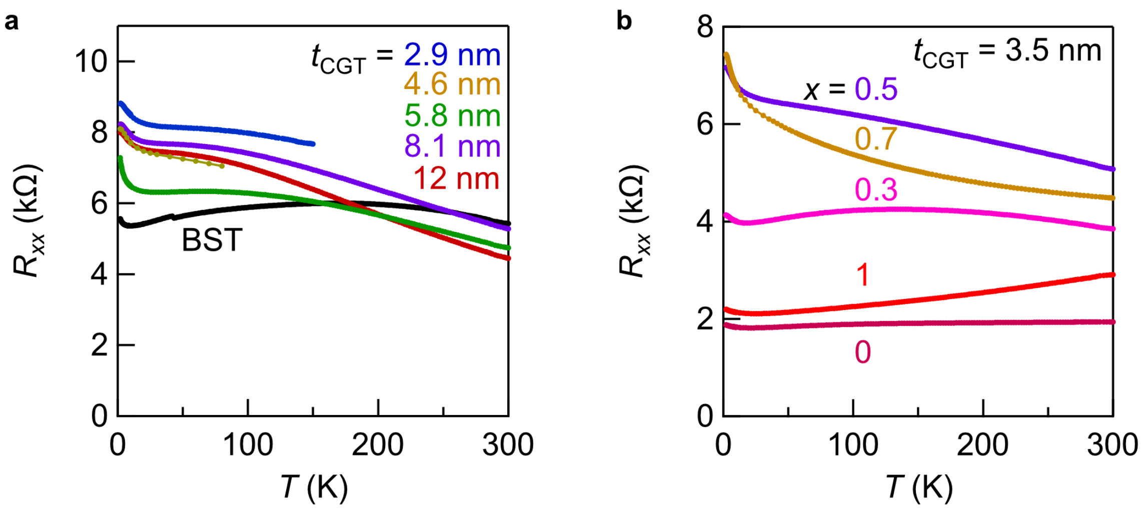

Supplementary Fig. 2 | Electrical resistance of CGT/BST bilayers. a,b, T dependence of R_{xx} in CGT/$(Bi_{1-x}Sb_x)_2Te_3$ (6 nm) bilayer films with various CGT thicknesses (t_{CGT}) (**a**) and Sb fraction (x) (**b**).

Supplementary Note 3 | Additional magnetization data of CGT/BST bilayers.

To see if the magnetic properties of the CGT layers with various thicknesses affect the magnetization switching behaviours, we conducted magnetic anisotropy measurements for the CGT/BST(x = 0.5) films with different CGT thicknesses (t_{CGT}) of 2.9 nm (Supplementary Fig. 3a) and 5.8 nm (Supplementary Fig. 3b), and also compared with the CGT single layer films which were investigated in our prior publication[2]. As shown in Supplementary Fig. 3c, while both the coercive force (μ_0H_c) and the anisotropy field (μ_0H_K) increase as t_{CGT} decreases, they show a saturating behaviour in the thinner CGT layer region (t_{CGT} < 6 nm). Thus, the impact of magnetic anisotropy change appears small for this regime. In the thicker region (t_{CGT} = 8.1 nm and 12 nm), on the other hand, it can be a possible origin of the increase of the switching efficiency as shown in Fig. 2e of the main text.

In addition, we show in Supplementary Fig. 4c the temperature dependence of μ_0H_K, which indicates a relatively weak temperature dependence compared with μ_0H_c elucidated from the out-of-plane (Supplementary Fig. 4a) and in-plane (Supplementary Fig. 4b) magnetization measurements.

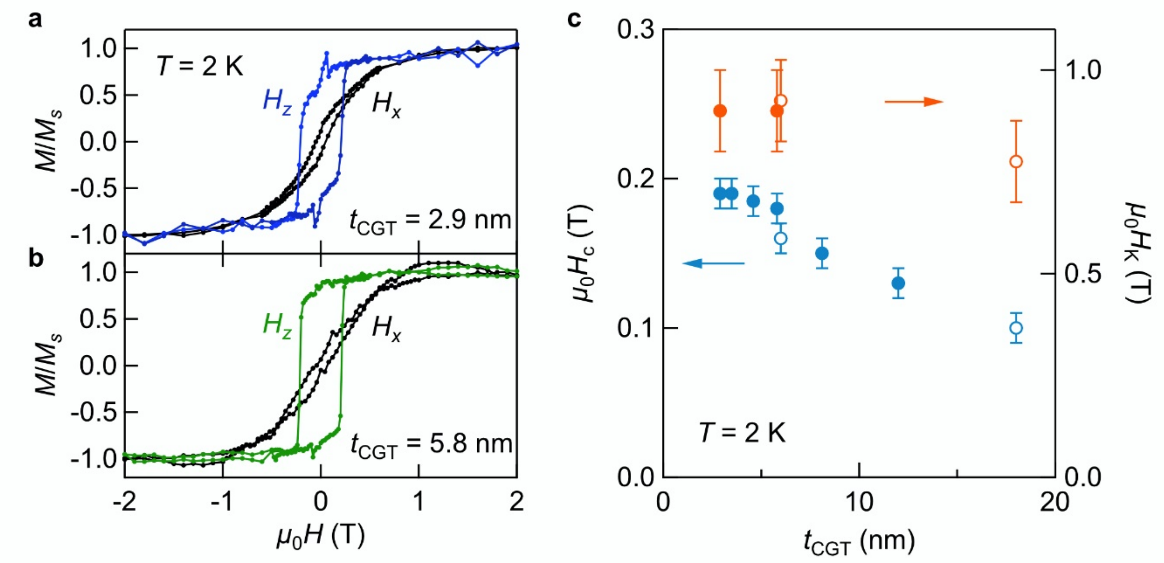

Supplementary Fig. 3 | Dependence of CGT thickness on magnetic anisotropy. a,**b**, Out-of-plane (coloured) and in-plane (black) magnetization hysteresis loops of the CGT(t_{CGT} = 2.9 nm (a), 5.8 nm (b))/BST(x = 0.5, 6 nm) heterostructures at T = 2 K. **c**, t_{CGT} dependence of the coercive field ($\mu_0 H_c$) (left vertical axis) and the anisotropy field ($\mu_0 H_K$) (right vertical axis). The filled and open circles indicate the CGT/BST heterostructures and the CGT single layers, respectively, where the result of the latter is adopted from Ref. S2. The error bars indicate measurement uncertainty.

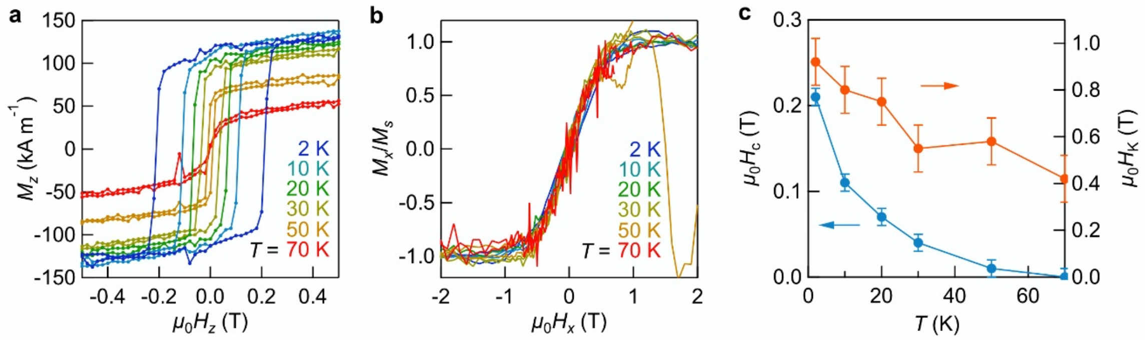

Supplementary Fig. 4 | Dependence of temperature on magnetic anisotropy. a,**b**, Out-of-plane (**a**) and in-plane (**b**) magnetization hysteresis loops of the CGT(t_{CGT} = 5.8 nm)/BST(x = 0.5, 6 nm) heterostructure at various T. **c**, T dependence of $\mu_0 H_c$ (left vertical axis) and $\mu_0 H_K$ (right vertical axis). The error bars indicate measurement uncertainty.

Supplementary Note 4 | Temperature estimation under current pulse injection.

To estimate the heating by current-pulse injection, we use the change of the coercive field upon current pulse injection. While changing the magnetic field (H_z), we inject current-pulses, where the spin-orbit torque induced magnetization switching does not occur in this magnetic-field direction condition. Then, the heating shrinks the H_c by the highest T reached with the current pulse injection and promotes the H-induced magnetization reversal (Supplementary Fig. 5b). We read the Hall resistance (R_{yx}) with low current excitation (J_x = 10 μA) after the sample was cooled again at each pulse. In Supplementary Fig. 5c, we scale the H_c with T (at a low current J_x = 10 μA) (Supplementary Fig. 5a) and the injected current density (at the base temperature of 2 K). From the T vs. J_x relationship, we can estimate the temperature increase by the current pulse injection, as shown in Supplementary Fig. 5d. At $j_x \sim 5$ A cm^{-1} ($J_x \sim 5$ mA), which corresponds to the switching current for t_{CGT} = 8 nm, 12 nm samples, T gets close to T_C (= 80 K) of the CGT layer. From this result, the observed reduction of the switching ratio R_{yx}^{sw}/R_{yx}^{AH}, shown in Fig. 2d, can be ascribed to the current-induced heating effect that some part of the CGT layer reaches the temperature higher than T_C, and thus cannot be fully reversed by the current injection.

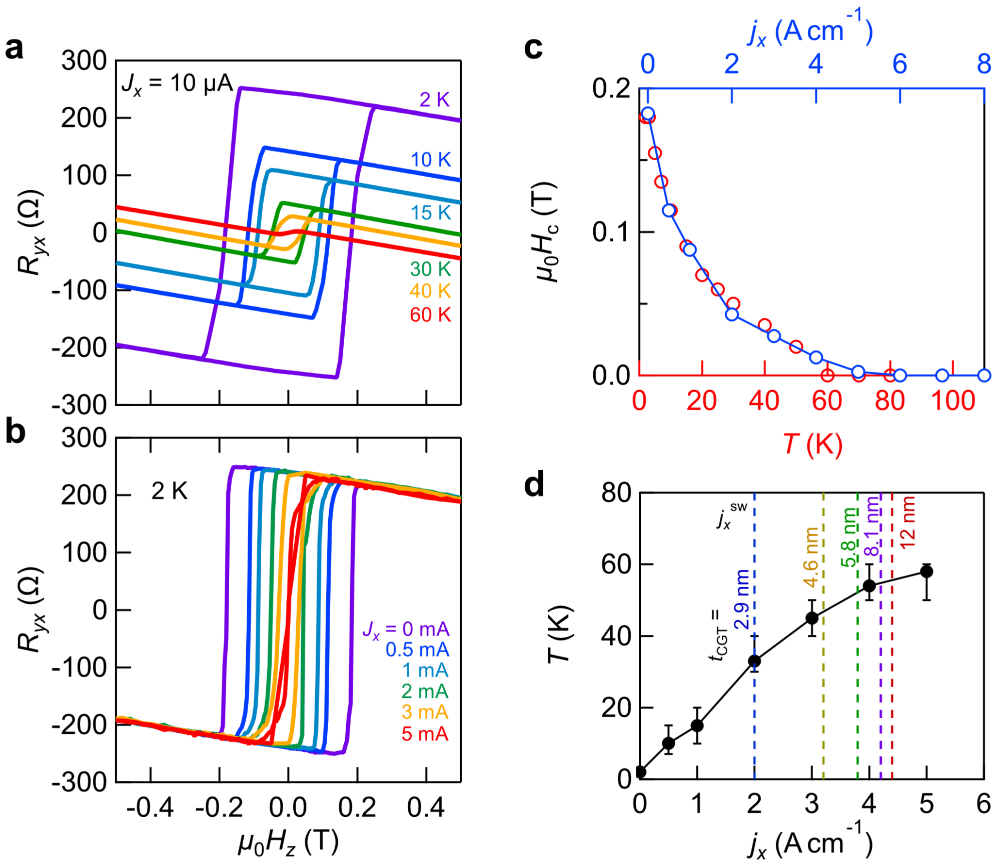

Supplementary Fig. 5 | Temperature estimation under current-pulse injection. a,**b**, Out-of-plane magnetic field ($\mu_0 H_z$) dependence of R_{yx} under current excitation of J_x = 10 μA measured at 2, 10, 15, 30, 40, and 60 K (a) and under current-pulses with amplitudes of J_x = 0, 0.5, 1, 2, 3, and 5 mA (b) at $\mu_0 H_x$ = 0 T in the CGT(t_{CGT} = 5.8 nm)/BST(x = 0.5, 6 nm) heterostructure. **c**, $\mu_0 H_\mathrm{c}$ plotted as a function of T (bottom axis) and pulse current density (j_x) (top axis). **d**, T estimation as a function of the pulse current density. The broken lines indicate the switching current density j_x^{sw} for the respective-thickness CGT samples. The error bars indicate uncertainty arising from comparing temperatures in (c).

Supplementary Note 5 | Magnetic domain structure of a CGT/BST bilayer.

We show the magnetic domain structure of a CGT(12 nm)/BST(x = 0.5, 6 nm) bilayer film in a virgin state after zero-field cooling observed by magnetic force microscopy (MFM). As shown in Supplementary Fig. 6, the typical size of the labyrinthine domain structure is smaller

than 1 μm (≪ 10-μm-wide Hall bars), which supports that the antidamping-like spin-orbit torques act on domain wall motion.

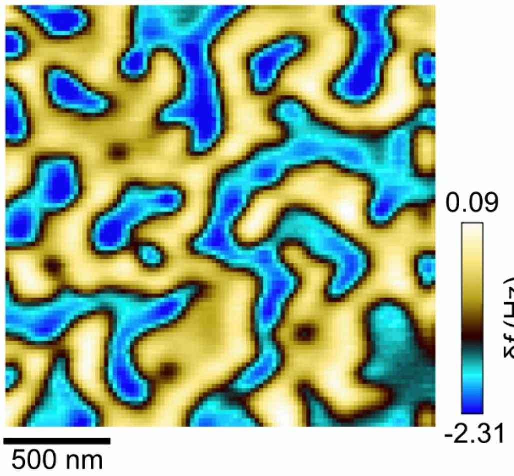

Supplementary Fig. 6 | Magnetic force microscopy image of a CGT/BST bilayer. MFM image of the 12-nm-thick CGT film on the 6-nm-thick BST/InP substrate. The MFM was carried out at T = 10 K in a scanning probe microscope (attocube AFM/MFM I). We used a MFMR cantilever (NANO WORLD) with a resonance frequency of ~ 76 kHz and a tip radius of ~ 50 nm. The measurement was performed with a cantilever excitation amplitude of 20 nm and a lift height of ~ 100 nm. The MFM signal, the change of the cantilever resonance frequency δf, is proportional to the gradient of the out-of-plane stray field from the sample.

Supplementary Note 6 | Second harmonic Hall voltage measurement in a CGT/BST bilayer.

We conducted the second-harmonic Hall voltage ($V_y^{2\omega}$) measurement[3,4] in a CGT/BST bilayer. We applied a magnetic field (H_x) parallel to the ac current direction (the current amplitude J_x = 10 μA and frequency ω = 13 Hz), where the second-harmonic Hall voltage is expected to be maximized. As shown in Supplementary Fig. 7, we observed the large value of the second harmonic resistance defined by $R_{yx}^{2\omega} = V_y^{2\omega}/J_x$, which is anti-symmetric with H_x and

pronounced at around the magnetic anisotropy field $H_K = 0.9$ T. In the scenario that the second harmonic Hall resistance is generated by the spin-orbit torques[3,4], where the out-of-plane component of the magnetic oscillation produces the anomalous Hall effect. The $R_{yx}^{2\omega}$ can be given by

$$R_{yx}^{2\omega} = -\frac{1}{2}\frac{R_{yx}^{\mathrm{AH}} H_{\mathrm{eff}}}{|H_x| - H_{\mathrm{K}}}, \quad (1)$$

where R_{yx}^{AH} stands for the out-of-plane anomalous Hall resistance. By fitting the result of Supplementary Fig. 7 with this formula, the effective field H_{eff} generated by the spin-orbit torques can be evaluated as 500 mT. Furthermore, this effective field gives the charge-to-spin current conversion efficiency

$$\xi^{\mathrm{SH}} = \frac{2e\mu_0 M_{\mathrm{s}} H_{\mathrm{eff}} t_{\mathrm{CGT}}}{\hbar j_x} \sim 1000\ \mathrm{nm}^{-1}, \quad (2)$$

where $j_x = J_x/W = 10^{-2}$ A cm^{-1} (W: the sample width 10 μm). This value by far exceeds that obtained by the current-induced switching experiments ($\xi \sim 1$ nm^{-1}).

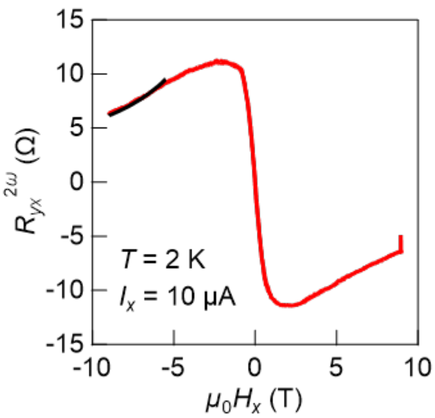

Supplementary Fig. 7 | Second-harmonic Hall resistivity. In-plane magnetic field ($\mu_0 H_x$) dependence of second-harmonic Hall resistivity for the CGT(t_{CGT} = 5.8 nm)/BST(x = 0.5, 6 nm) with the current of 10 μA along the magnetic field direction (x). The black line indicates the fitting curve from Eq. (1).

We have encountered such an unphysical situation also in studies on the magnetic

topological insulator Cr-doped BST/BST (Ref. 5) and the bulk Rashba semiconductor (Ge,Mn)Te (Ref. 6); to account for this, we have argued that the second-harmonic signal can appear without the spin-orbit torque induced magnetization oscillation. In the papers, we discuss that the second harmonic Hall resistance can include another significant scattering process, where the spin-momentum locked electrons are asymmetrically scattered by magnons generated in the magnetic layer. The transverse electron scattering under the conservation of spin-angular momentum via the magnon emission and absorption processes gives rise to a J_x^2-proportional voltage (or second harmonic voltage) along the transverse direction. Thus, we believe that this method is inappropriate to accurately evaluate the efficiency in the present TI-based materials system. Nevertheless, such a nonlinear Hall response due to the magnon scattering can provide another concrete evidence for the spin-momentum locked surface Dirac electron dynamics coupled to the adjacent ferromagnetic layer.

Supplementary Note 7 | Temperature dependence of current-induced magnetization switching.

We conducted the T-dependent current-induced switching experiments in the CGT(t_{CGT} = 5.8 nm)/BST(x = 0.5, 6 nm), in which finite spontaneous magnetization is seen up to 50 K (Supplementary Fig. 8a). From the results of the current-induced switching experiments (Supplementary Fig. 8b) and the magnetization measurements (Supplementary Fig. 8c), we extract the switching efficiency coefficient ξ [$= 2e\mu_0 M_s H_c t_{\mathrm{CGT}}/(\hbar j_x^{\mathrm{sw}})$] and the switching ratio $R_{yx}^{\mathrm{sw}}/R_{yx}^{\mathrm{AH}}$ (Supplementary Fig. 8d). While the switching is accomplished at all the temperatures below 50 K as seen in the nearly constant $R_{yx}^{\mathrm{sw}}/R_{yx}^{\mathrm{AH}}$, the ξ sharply increases with decreasing the temperature. We speculate that the enhancement of ξ is due to the enhancement of magnetic proximity coupling which is evident in the sharp increase of the anomalous Hall resistance (R_{yx}^{AH}) shown in Supplementary Fig. 8c, rather than the moderate increase of the spontaneous magnetization density (M_s). The enhanced proximity coupling makes the coupling

between the surface state spins and localized spins strong and could result in the more efficient spin-orbit torque switching.

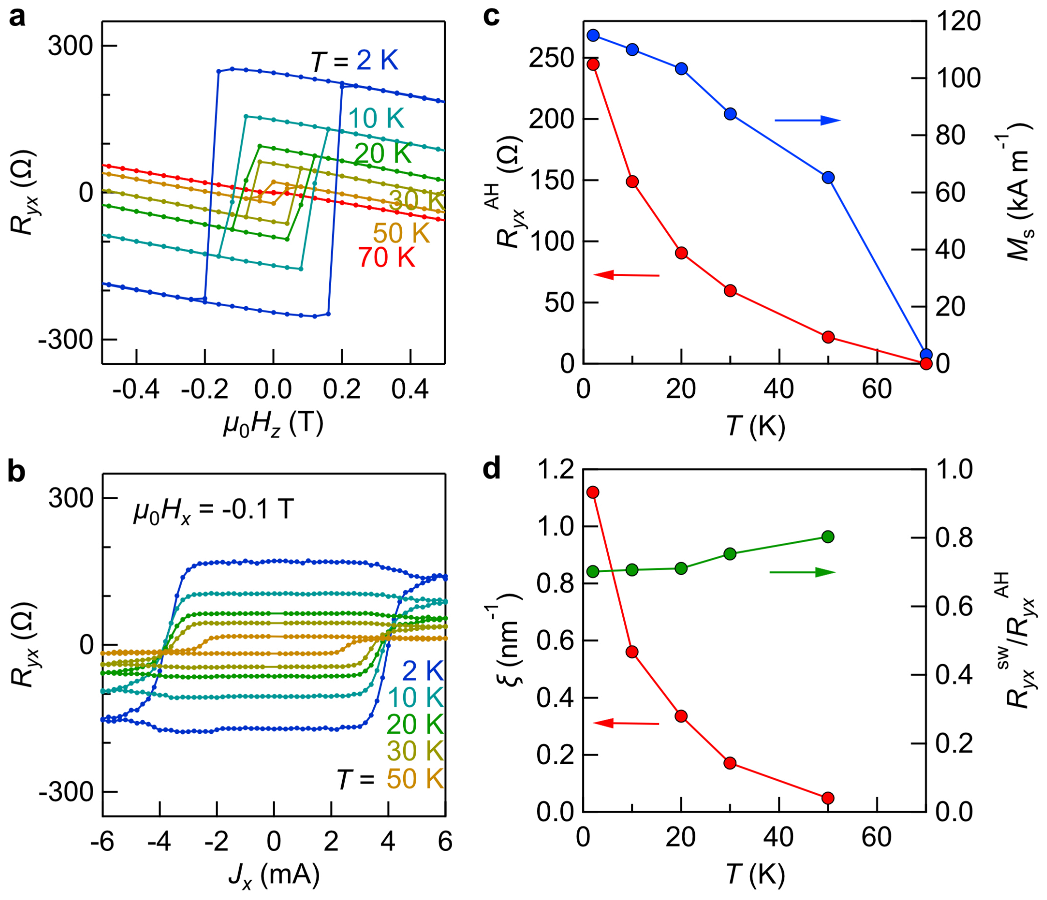

Supplementary Fig. 8 | Dependence of temperature on magnetization switching. **a**, $\mu_0 H_z$ dependence of R_{yx} in the CGT(t_{CGT} = 5.8 nm)/BST(x = 0.5, 6 nm) at various temperatures (T = 2, 10, 20, 30, 50, and 70 K). **b**, Magnetization switching in the CGT/BST devices at various temperatures (T = 2, 10, 20, 30, and 50 K) under $\mu_0 H_x$ = −0.1 T. **c**, $\mu_0 H_z$ dependence of the magnetization M_z. **d**, T dependence of the coefficient ξ [= $2e\mu_0 M_s H_c t_{CGT}/(\hbar j_x^{sw})$] (left vertical axis) and the switching ratio of R_{yx}^{sw}/R_{yx}^{AH} (right vertical axis).

Supplementary References

1. Mogi, M. et al. Large anomalous Hall effect in topological insulators with proximitized

ferromagnetic insulators. *Phys. Rev. Lett.* **123**, 016804 (2019).

2. Mogi, M. et al. Ferromagnetic insulator $Cr_2Ge_2Te_6$ thin films with perpendicular remanence. *APL Mater.* **6**, 091104 (2018).
3. Kim, J. et al. Layer thickness dependence of the current-induced effective field vector in Ta|CoFeB|MgO. *Nat. Mater.* **12**, 240-245 (2013).
4. Garello, K. et al. Symmetry and magnitude of spin-orbit torques in ferromagnetic heterostructures. *Nat. Nanotech.* **8**, 587-593 (2013).
5. Yasuda, K. et al. Current-nonlinear Hall effect and spin-orbit torque magnetization switching in a magnetic topological insulator. *Phys. Rev. Lett.* **119**, 137204 (2017).
6. Yoshimi, R. et al. Current-driven magnetization switching in ferromagnetic bulk Rashba semiconductor (Ge,Mn)Te. *Sci. Adv.* **4**, eaat9989 (2018).
